# Effects of extreme events on land-use-related decisions of farmers in Eastern Austria: the role of learning

**DOI:** 10.1007/s13593-023-00890-z

**Published:** 2023-05-12

**Authors:** Claudine Egger, Andreas Mayer, Bastian Bertsch-Hörmann, Christoph Plutzar, Stefan Schindler, Peter Tramberend, Helmut Haberl, Veronika Gaube

**Affiliations:** 1 Department of Economics and Social Sciences, Institute of Social Ecology, University of Natural Resources and Life Sciences, Schottenfeldgasse 29, 1070 Vienna, Austria; 2 Environment Agency Austria, Spittelauer Lände 5, 1090 Vienna, Austria; 3 Community Ecology and Conservation, Faculty of Environmental Sciences, Community Ecology and Conservation Research Group, Kamýcká 129, CZ-165 00 Prague 6, Czech Republic

**Keywords:** Agent-based modeling, Learning, Scenario analysis, Adaptation, Austria, Agriculture

## Abstract

European farm households will face increasingly challenging conditions in the coming decades due to climate change, as the frequency and severity of extreme weather events rise. This study assesses the complex interrelations between external framework conditions such as climate change or adjustments in the agricultural price and subsidy schemes with farmers’ decision-making. As social aspects remain understudied drivers for agricultural decisions, we also consider value-based characteristics of farmers as internal factors relevant for decision-making. We integrate individual learning as response to extreme weather events into an agent-based model that simulates farmers’ decision-making. We applied the model to a region in Eastern Austria that already experiences water scarcity and increasing drought risk from climate change and simulated three future scenarios to compare the effects of changes in socio-economic and climatic conditions. In a cross-comparison, we then investigated how farmers can navigate these changes through individual adaptation. The agricultural trajectories project a decline of active farms between −27 and −37% accompanied by a reduction of agricultural area between −20 and −30% until 2053. The results show that regardless of the scenario conditions, adaptation through learning moderates the decline in the number of active farms and farmland compared to scenarios without adaptive learning. However, adaptation increases the workload of farmers. This highlights the need for labor support for farms.

**Supplementary Information:**

The online version contains supplementary material available at 10.1007/s13593-023-00890-z.

## Introduction

Climate change threatens agricultural production not only through changing climatic conditions such as further increasing temperatures and changing rainfall patterns (Loarie et al. 2009; Box et al. 2019) but also through severe extreme weather events (Zwiers et al. 2013; Stott 2016; Diffenbaugh et al. 2017; IPCC 2022), such as droughts, heavy rainfall, or floods (Otto et al. 2018; Mukherjee et al. 2018; Trenberth 2018) that cause soil erosion (Borrelli et al. 2020) and yield declines (Vogel et al. 2019). Human impacts on global ecosystems increased the frequency of severe extreme events worldwide (Stott 2016; Ummenhofer and Meehl 2017; IPCC 2022), which are also an emerging topic in science: While the first Bulletin of the American Meteorological Society, published in 2012, covered the occurrence of six extreme events (Herring et al. 2014), the most recent publication not only covers a multitude of extreme events but also focuses on their anthropogenic causes and effects (Herring et al. 2022). In contrast to the long-term impacts of climate change, of which the full extent will only become apparent in the medium to long-term future, extreme weather events have an immediate impact on regional agricultural and socio-economic systems (Gobiet and Truhetz 2008). This makes it essential to better understand the societal coping strategies of farmers to such events. Because occurrence and magnitude of extreme events rise (Stott 2016; IPCC 2022), private and public decision-making is increasingly challenged to develop suitable response strategies. The damage potential of extreme events depends not only on their physical characteristics, but crucially also on the response of the affected socio-ecological systems and the ability to adapt and transform through feedback mechanisms (Carpenter et al. 2012). The ability of actors (individuals and organizations) to overcome risks (e.g., food scarcity) imposed by changes in the natural environment through adaptation to new circumstances has been a key success factor for societal development ever since the hunter-gatherer age (Boserup 1965).

Climate change poses long-time risks to agricultural production through the gradual change in temperature and precipitation and the risks of intermediate losses through the rising frequency and severity of extreme events (IPCC 2022). Depending on the governance level, there are different, complementary response strategies to address these risks. (1) Reduce climate change in a collective societal effort, e.g., through mitigation attempts that limit the temperature rise compared to the pre-industrial level to 1.5°C (Newell et al. 2021). (2) In addition, farmers can pursue individual reaction strategies by adapting their decision-making as response to short-term changes such as yield loss due to extreme events, and thereby anticipating changing agricultural conditions from a long-ranging changing climate. This requires that farmers perceive climate change as risk factor that needs to be considered in their decision-making, and several studies identify a relation of climate change perception and related risk association (Osbahr et al. 2011; Arbuckle et al. 2015; van Winsen et al. 2016; Schattman et al. 2016; van Duinen et al. 2016; Shukla et al. 2019; Mitter et al. 2019; Zagaria et al. 2021).

Brown et al. (2017) stress that the decisions on adaptation to climate change are shaped by social, economic, environmental, and political contexts. The influence of social factors such as gender or social relations in agricultural production systems remains understudied (Davidson 2016), leaving significant gaps in related literature contributions on farmers’ decision-making in relation to climate change adaptation. The dominant assumptions of economic rationality lack full apprehension of social complexity and thereby limit the potential for integrating realistic forms of decision-making into land-use-based adaptation models (Groeneveld et al. 2017; Brown et al. 2017). Many agricultural land-use models that focus on climate change adaptation do not include any form of learning (Brown et al. 2017), meaning that the integration of learning in a functional way in which the experience of a changing environment results in adaptation (De Houwer et al. 2013). Similarly, Huber et al. (2018) identified learning as underrepresented aspect in a review on European land-use models. In cases where learning is integrated in land-use models, the focus lies on the integration of social learning based on network experiences or beliefs but learning from an individual perspective has rarely been studied (Brown et al. 2017).

In order to investigate spatially explicit effects of individual decision-making, agent-based modeling represents an approach that is particularly suitable. While system-dynamic models follow a “top-down” approach in which changes of a socio-ecological system emerge based on the overarching systemic relationships, agent-based models (ABMs) simulate systemic transitions in a “bottom-up” process that starts with the individual agent. ABMs consider interactions and the feedback dynamics emerging from agent’s reaction to changes in their social and biophysical environment when simulating the dynamics of a system (Verburg et al. 2016). This predestines such models for the analysis of socio-ecological systems such as farming systems, as they can integrate the complex interrelations between land-use decision-making and changes in external socio-economic, biophysical, and climatic conditions (Verburg et al. 2019).

Recent agent-based applications that integrate climate-related risks (van Duinen et al. 2016; Amadou et al. 2018; Zagaria et al. 2021) primarily focus on peer influence from social networks. In combination with the agent’s goal orientation towards profit maximization, these models consider learning in a social and economic context but neglect other factors such as political or environmental impacts. While the influence of stochastic extreme events on land-use management has been integrated in ABMs (Mayer et al. 2022; Egger et al. 2022), the occurrence of extreme events as driver for individual learning has primarily been modeled for drought perception and the subsequent transition to irrigated production systems (van Duinen et al. 2016; Yang et al. 2020; Zagaria et al. 2021). With this study, we want to deepen the knowledge on learning from extreme events related to climate change.

As European farmers operate within boundaries set by socio-political guidelines such as CAP policies and their climatic environment (Stoate et al. 2009; Peltonen-Sainio et al. 2010; van Zanten et al. 2014; Pe’er et al. 2017), this paper investigates how farm households can navigate changes coming from their socio-ecological framework conditions through individual adaptation investigating the following questions:

- How do climatic and socio-economic scenarios impact the agricultural development trajectories and how do farms respond to climate change impacts and changes in agricultural subsidy schemes?
- How does the integration of “learning” triggered by extreme events affect the development of farms?
- How can individual farms adapt to external framework changes? Which socio-economic effects does this have on farms?

**Fig. 1:**
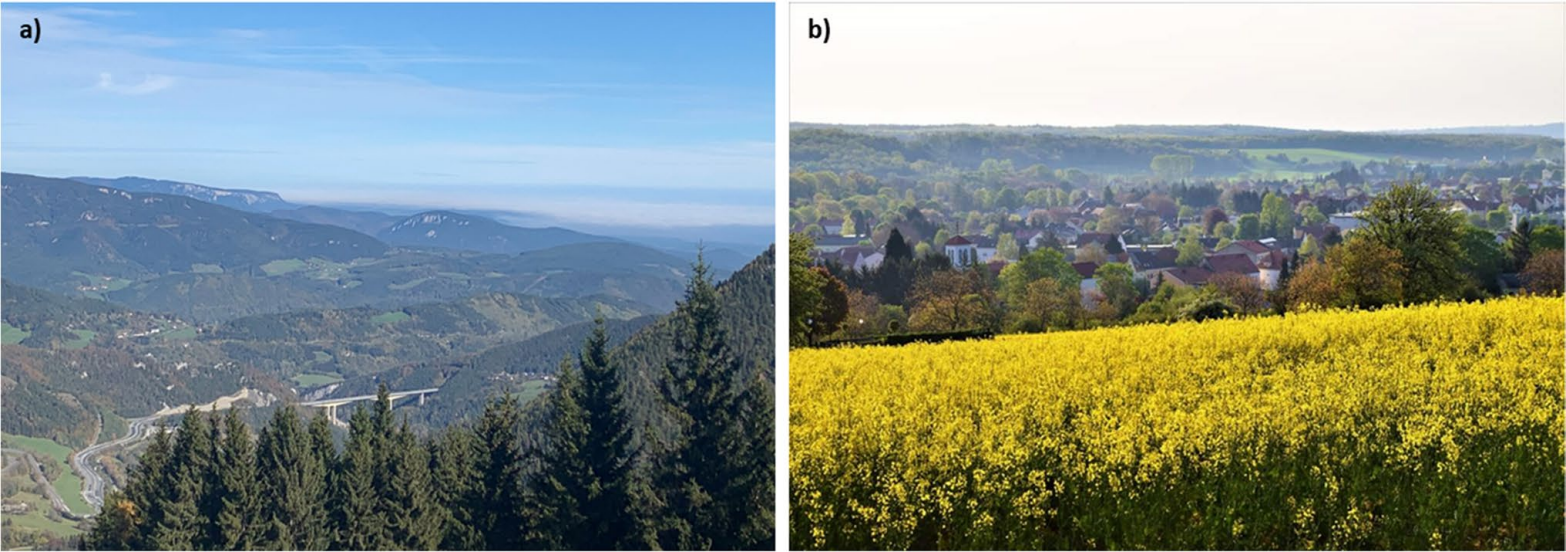
**a** View into the study region from the Mountain Semmering: showing the transition from the mountainous West towards the lowlands in the Eastern parts. **b** View over a rapeseed field in the Eastern lowlands (Oberpullendorf).

We use the SECLAND ABM (Mayer et al. 2022; Egger et al. 2022) to explore the socio-ecological effects of adaptation within the existing agricultural production system through the application of on-site farm improvement management actions. We apply the model to a study region in Eastern Austria and simulate agricultural trajectories for the years 2015–2053 in line with three distinct future scenarios to evaluate the impact learning from extreme events has on agro-structural development. The study site, characterized by cropland farming in the Eastern lowlands and livestock farming in the hilly areas of the Western parts (Fig. 1), belongs to a region in Austria predicted to experience noticeable impacts of climate change in the upcoming decades (Haslmayr et al. 2018).

## Methods

### Study region

**Fig. 2:**
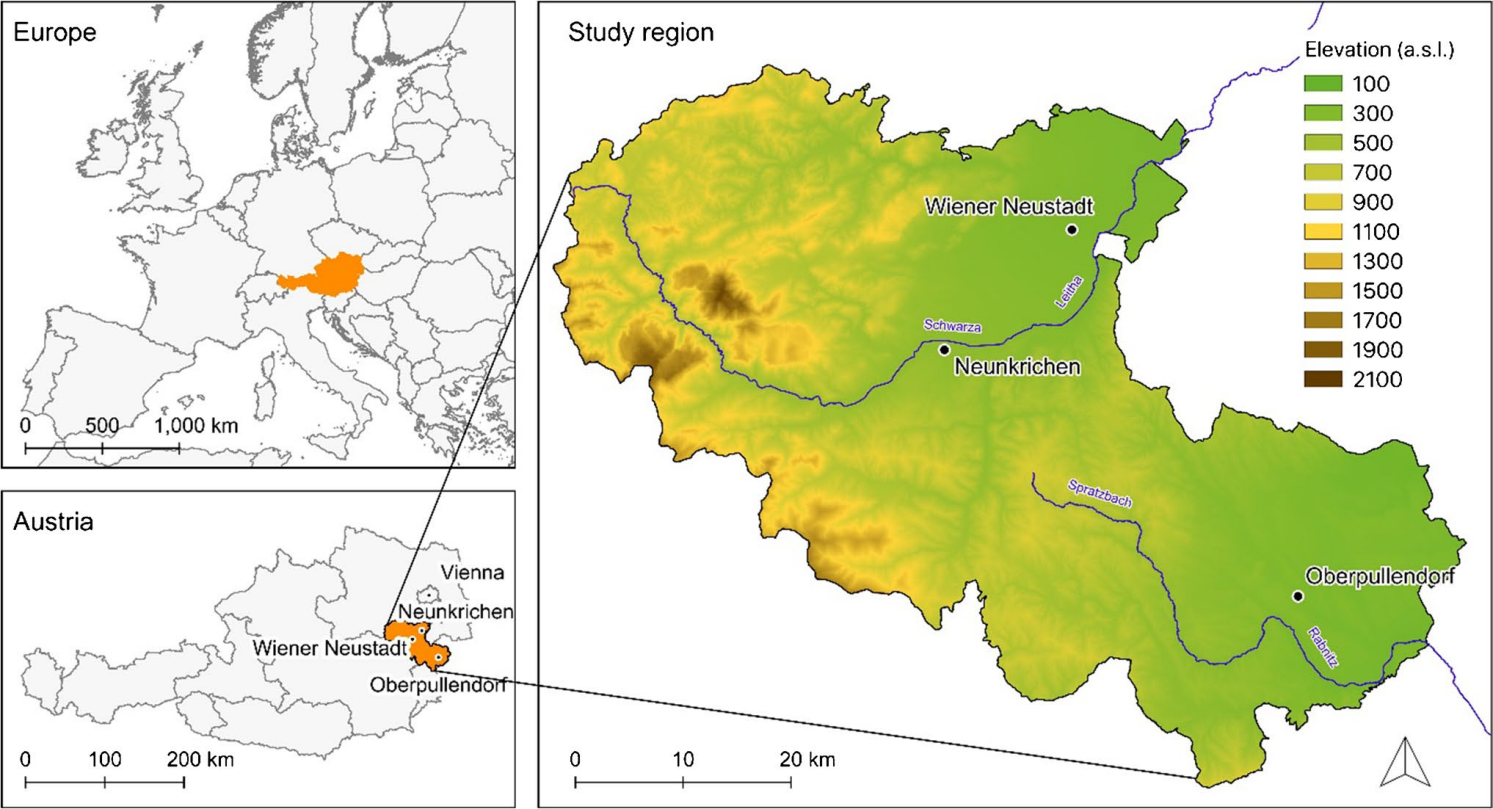
Location of the study region encompassing the districts Neunkirchen, Wiener Neustadt Land, and Oberpullendorf in the Eastern part of Austria (Natural Earth 2022).

The study region consisted of the three districts Oberpullendorf, Neunkirchen, and Wiener Neustadt Land located in Eastern Austria (Fig. 2). The area encompasses a total area of about 2.818km^2^, 107 municipalities, and a population of about 203,000. Biogeographically, the study region is divided: the West situated on the outskirts of the Alps belonging to the Alpine and the Eastern parts being part of the Continental region (EEA 2017). Elevation ranges from 169 (Northeast, Southeast) to 2056 m a.s.l (Northwest). Correspondingly, the annual average precipitation is between 400 and 2000 mm/year, and average annual temperatures range from +4° to +12° (Hiebl and Frei 2016). Farmers in the study region are facing rising risks of weather extremes, especially droughts, that can hardly be compensated through irrigation because water availability is generally limited, and small-scale farming prevails profitable irrigation. Farmers reported that the recurring droughts and the lack of precipitation already had negative impacts on agricultural production quantity (e.g., deficits in harvested grassland biomass, lower crop yields) and quality (e.g., of summer grains) (Fessler 2021; Petz 2021).

### Conceptual modeling framework

**Fig. 3:**
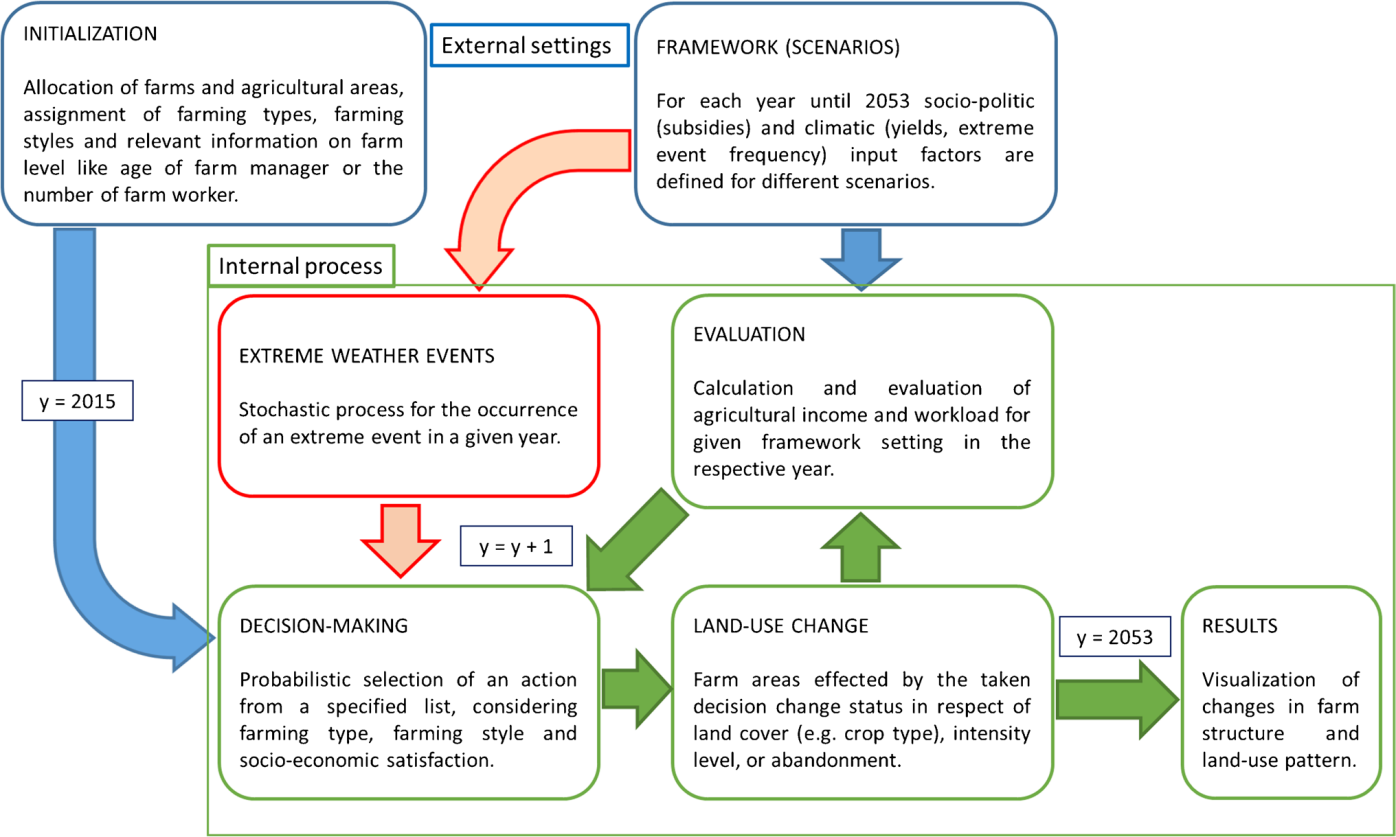
Overview of the modeling process and functional relations between external settings (blue), internal dynamics (green), and the effects of extreme weather events (red) of the SECLAND ABM.

The purpose of the SECLAND ABM (Egger et al. 2022) is the study of systemic feedbacks between external changes in the socio-political, biophysical, and climatic framework conditions and the internal dynamics arising from the decision-making of farm agents and its effects on the land-use dynamics of the study region (Dullinger et al. 2020; Mayer et al. 2022). This application of the model features the integration of *learning* as model update that builds upon the interactions between the decision-making process and the occurrence of extreme weather events. Figure 3 depicts an overview of the model’s structural relations between external and internal dynamics. A thorough description of the SECLAND model is available in Dullinger et al. (2020), Egger et al. (2022), and Mayer et al. (2022). The ODD description (Müller et al. 2013) of the here presented updated version of the model can be found in the supplementary material (Table SI2).

### Initialization

#### Farms and agricultural area

The ABM is initialized with 2990 farms that cultivate 76,220 patches (parcels of land) with a total area of 87,545 hectares (ha) agricultural land in 2015. Farm agents are distinguished according to the following core characteristics: farming type, farming style, and production system. There are three farming types: cash crop farms, processing farms that engage either in pig fattening or suckler production, and (cattle) livestock farms focusing on meat or milk production (for an extensive description see Supplementary Tables SI2 and SI4). The farming style (Schmitzberger et al. 2005) represents the “value system” of farmers. The model incorporates five farming styles that are decisive for the decision-making of farmers: “innovative” (highly flexible and open to learning), “yield optimizer” (prioritizes yield maximization), “support optimizer” (tries to maximize income from subsidies), “idealist” (self-realization in farm work), and “traditionalist” (continuing the tradition). Without specific information on proportions, farming styles are randomly assigned among the model’s farmer population. Farm management is either conventional or organic, which each includes five intensity levels that differ in labor demand, yields, prices and costs, respectively, gross margins, and subsidies. Farmland consists of croplands and/or grasslands. Cropland is managed in three cropping cycles with sugar beet, rapeseed, and soy as lead crops. Grassland contains meadows, pastures, and mountain pastures. Cattle livestock units (lu) are bound to grassland. Livestock numbers are computed based on the forage provision (computed as area × intensity level) of meadows/pastures held by a farm. Porcine livestock numbers are assigned to farms (in lu) (see Supplementary Table SI1).

#### Input data

For the setup of the ABM, we relied on the complementary combination of quantitative and qualitative data. The main quantitative source was the Invekos (IACS) data that we used for the initialization of farm structure. Farm areas were based on spatially explicit land-use information on parcels provided by the Invekos GIS-data. The initialization year 2015 was the first year with comprehensive, parcel-specific land-use information. We chose the simulation period 2015–2053 as such to allow subsequent computations on crop cultures as moving average for the year 2050. Subsidies, farm, and livestock classification, numbers, and densities (e.g., livestock units per hectare, lu/ha) are drawn from the aggregated analysis of reported Invekos numbers. We relied on production-region-related yield data from the bookkeeping farms (*Buchführungsbetriebe*). For missing (either unreported or database secrets) values and to compute gross margins, we used price and cost and labor requirement information on state/federal level (BAB 2021) and for socio-economic inputs (farmers age, off-farm income, etc.) data from Statistik Austria (see Supplementary Tables SI3–SI5).

We used qualitative data to understand farmers’ motivations and relevant management actions, which is a common approach in agent-based modeling (Janssen and Ostrom 2006; Valbuena et al. 2008; Smajgl et al. 2011). We conducted semi-structured interviews with 20 farmers (11 cash crop and 9 livestock farms; Fessler 2021; Petz 2021) during summer 2020 to identify and calibrate action sets with probabilities. In addition, we held an online stakeholder workshop with regional experts and decision-makers in July 2020, to discuss past and current agricultural trends, the role of climate change-related extreme events (recurring droughts and heat waves) and assess the scenario storylines on future development of agriculture. The workshop was followed up by two expert interviews, where important topics that emerged during the workshop were discussed in more detail (see Supplementary Table SI6).

### Internal processes

#### Evaluation indicator and decision-making

Farms in the SECLAND ABM are setup as bounded rational agents (Groeneveld et al. 2017) that pursue well-being rather than profit maximization. We defined well-being as satisfying balance between (minimum) income and (maximum) workload (per fulltime farm worker (full time equivalent, fte)). Farms invest labor to cultivate their patches (related crops and livestock units) from which they generate agricultural income. Farm intensity levels are linked with specific labor input demand and gross margin outputs. Farms use three satisfaction criteria to annually evaluate their well-being: (1) a minimum agricultural income (per fte) that is based on reported averages per farm type (Grüner Bericht NÖ 2017; SI2). (2) A workload maximum of 1800 h (per fte) that relates to the yearly workload of Austrian employees (WKO Statistik 2020). (3) To account for income comparison among peers, the hourly agricultural income (computed per farming type) needs to exceed a yearly computed minimum (average value - 1SD; see Supplementary Table SI2). Depending on the satisfaction (with either or both), farms take a decision among ten possible actions that affect their farm management to improve/maintain satisfaction by increasing/decreasing their workload and/or income (Table 1). Based on the synthesis of the qualitative data, all relevant actions were assigned with pre-defined probabilities that depend on income-workload satisfaction, farming type, and farming style.

**Table 1:** Set of possible land-use decisions for individual farmers in SECLAND. Farms take land-use decisions based on their satisfaction with income and workload; probabilities depend on happiness, farm type, farming style and scenario.

Actions	Description
Act1: Nothing	Farms decide not to change their operations and postpone their decision into the next year
Act2: Hire farm worker	Farms hire an additional farm worker (10% of a full-time equivalent or 180 hours yearly)
Act3: Intensification	Farms with intensity level ≤ 4 increase their intensity level by +1
Act4: Extensification	Farms with intensity level > 1 decrease their intensity level by−1
Act5: Organic production	Farms switch their production style from conventional to organic, this is possible once in the 37 years simulation period
Act6: Land-use change	Farms decide between income gain (maximum income) or time saving (minimal time use) and switch their cropland between crop cycles accordingly
Act7: Afforestation	Farms plant forest on one of their grassland patches and decrease their suffer-counter
Act8: Expansion	Farms try to acquire 1 patch from the rental market to expand their farming size
Act9: Reduction	Farms dismiss additional workers if they are employed;
	Farms send 1 patch to the rental market to reduce their farming size
Act10: Termination	Farms pass their remaining areas to the rental market; patches change their patch unit to the one of the rental markets and set their intensity level to 0;
	Farms then track their “death” year and reason (termination) before switching their activity status to 0

#### Adaptive decision-making

Based on strategies from interviews with regional farmers (Fessler 2021; Petz 2021) as well as from previous projects in Austria (Freudenberg 2017; Perzl 2021) and France (Mayer et al. 2022), we identified four actions relevant for adaptation: land-use change, organic production, extensification, and expansion. For example, farmers mentioned that due to climate change, yield fluctuations are increasing and they need to compensate for this by expanding their farm size. We decided against the introduction of new actions, as these would have had the potential to introduce new risks, as for example irrigation often presented as adaptation measure to stabilize water supply for agricultural production (Wheeler et al. 2013; van Duinen et al. 2016; Zagaria et al. 2021) induces risks such as soil salinization (Singh 2021). A stakeholder workshop (Supplementary Table SI6) in the study region and expert interviews confirmed that water scarcity and decreasing ground water levels are already a problem for the region and would be amplified by irrigation projects. Therefore, we focused on the integration of adaptation strategies considering on-site farm measures within the existing set of actions. The results of this study, however, can give indication for the integration of new actions and decision-options in future model versions.

**Fig. 4:**
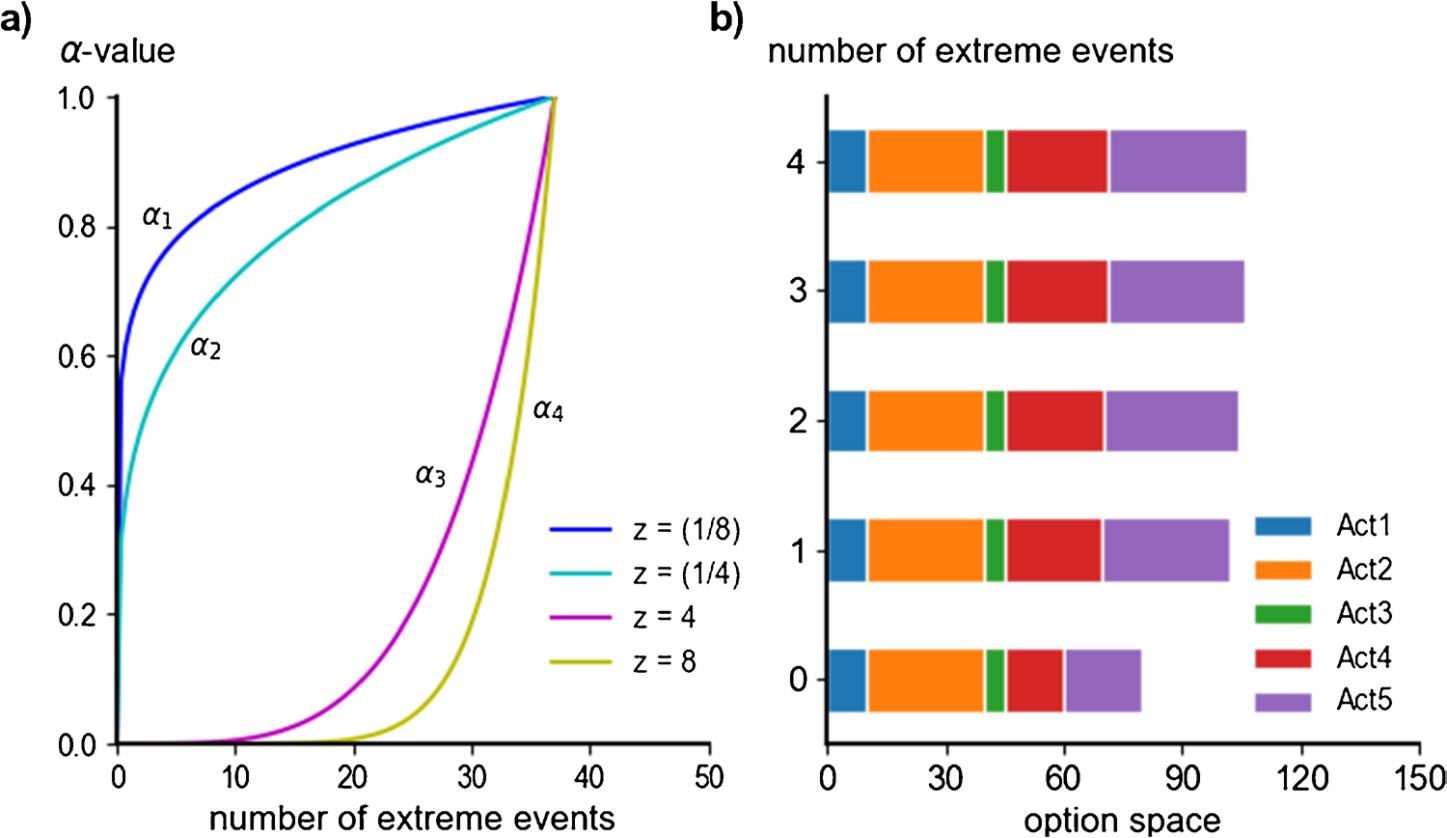
**A** Functional relationship of ${\alpha }_{i}$ for the adaptation types 1–4: the increasing value for the square root *z* reflects the increasing the resistance (inertia) towards adaptation; each occurrence of an extreme event *x* has a persisting effect on ${\alpha }_{i}$. **b** Illustrative visualization of the effects of ${\alpha }_{1}$ on decision-making: ${\alpha }_{1}$ increases the likelihood for the actions Act_4_ and Act_5_ with the rising number of extreme events *x* (for *x* 0–4).

In a recent study, Mitter et al. (2019) developed a typology for farmers’ attitudes towards adaptation strategies for a case study in Austria that builds upon their climate change perception and related climate risk association. They identified four types of farmers’ attitudes (climate change adaptors, integrative adaptors, cost-benefit calculators, and climate change fatalists) that were determined by farmers’ perception and appraisal of climate change as well as individual and regional farm characteristics. Depending on their respective attitudes, they show varying grades of inertia towards adopting climate change adaptation measures. To integrate adaptive decision-making in the SECLAND ABM, we decided to link four adaptation types, based on the distinction made by Mitter et al. (2019), with the model’s five farming styles. Schmitzberger et al. (2005) classify the farming styles according to their agricultural decision-making, ranking traditionalists as very static, idealists as static, support and yield optimizers as equally dynamic, and innovative as very dynamic. Based on this assessment, we associated the farming styles with adaptation types, for which we developed risk-related adaptation functions: with a presumable impact on the decision-making of innovative, lighter and equal effects for both optimizer types but minor effects on idealists and even less on traditionalists (Fig. 4a). This is represented via adaptation function:

$${\alpha }_{i}(x)={(x/37)}^{z}$$

where *i* denotes the four adaptation types 1–4 and *α*_i_ depends on the number of extreme events *x*. Each occurrence of *x* increases *α*_i_ persistently. *α*_i_ reaches its maximum of 1 in the (unlikely) case of 37 extreme events, meaning that one event occurred in each year of the modeling period. The root value *z* symbolizes the inertia towards change; the higher its value the lower is the willingness to incorporate adaptation (see Fig. 4a).

The *α*-functions affect the likelihood LAct_k_ of the aforementioned actions Act_k_ (*k*=4, 5, 6, 8) in the decision-making process, as their algorithm is altered by 1 + *α*_i_ (Fig. 4b). In the case of ${\alpha }_{i}$ reaching its maximum value of 1 (37 extreme events), the likelihood doubles. LAct_k_(*α*_i_) reflects the new likelihood of an action after the multiplication by (1+*α*_i_).

$$L{Act}_{k}\left({\alpha }_{i}\right)={LAct}_{k}*(1+{\alpha }_{i})$$

### External settings

#### Scenarios

Farms are exposed to several risks such as environmental, production, market, price, and political risks (Bard and Barry 2001; Huber et al. 2022). Subsidy schemes as well as market demand for agricultural products and prices are external factors in the SECLAND model. While market demand and price play a subordinate role for this application, we assess the variation of subsidy schemes in two distinct scenarios and focus on the model integration of *production risk* as result of climate change-induced extreme weather events.

As a result of the stakeholder process, we have outlined three distinct scenarios to assess future agricultural development pathways for the study region (Table 2). As baseline, a business-as-usual (BAU) scenario depicted the continuation of current development trajectories with constant subsidies and moderate impacts of climate change. In a high subsidy (HS) scenario, subsidies were used as a steering mechanism to target favorable socio-economic conditions for agricultural production. It describes high societal market intervention to achieve climate goals and, correspondingly, low impacts from climate change. In contrast, the free market (FM) scenario depicted the development of a society that forgoes controlling intervention and abolishes all subsidies by 2052 to focus on free market competition instead, which we combined with more pronounced climate change.

**Table 2:** Assumptions made to construct the scenarios driving the agent-based model: yields, prices, subsidies, preferences, and the probability of extreme events per annum (p.a.) distinguishing BAU (business-as-usual), HS (sustainability driven high subsidy), and FM (free-market & fast climate change) scenarios. Thresholds for workload and agricultural income are assumed to remain constant.

Scenarios	BAU	HS	FM
Yields	Decrease: 5–25%	Decrease: 0–10%	Decrease: 15–50%
Prices	Constant	conv: +15% org: +25%	Constant
Variable costs	Constant	Constant	Constant
Subsidies	Constant	Increase: conv. 50%/org. 75% in 5-year intervals until 2030, thereafter constant	Decrease: conv./org. 100% in 5-year intervals until 2030, thereafter constant
Workload	Increase: 10%	Increase: 5%	Increase: 10%
Extreme events	Expected value: 10% p.a. (random normal distribution, SD 2.5%) yields: decrease 40%	Expected value: 7.5% p.a. (random normal distribution, SD 2.5%) yields: decrease 30%	Expected value: 15% p.a. (random normal distribution, SD 2.5%) yields: decrease 60%
Alterations in farmer’s decision matrices	Constant	Constant	Constant, possibility to switch from org. to conv. production

The effects of climate change were accounted for in two ways in the model and the scenarios (Mayer et al. 2022; Egger et al. 2022). For the effect of climate change on yield development, we used region-specific yield prognoses for low (HS), moderate (BAU), and a large temperature increase (FM) from (Haslmayr et al. 2018) for the forecast of yields. Extreme weather events related with climate change that lower the annual harvest also played a crucial role in the modeling framework. While recent climate projections foresee rising intensity of such events for the study region, substantial differences based on climate scenarios are not expected until the second half of the twenty-first century (Gobiet and Kotlarski 2020). We therefore integrated stochastic weather extreme events with minor variations in severity and occurrence among the scenarios (Table 2).

### Limitations and model evaluation

We relied on random distributions to bridge data gaps. We performed 100 Monte Carlo (MC) runs per scenario to take into account the stochasticity of these assumptions, but remain within the limits of our computer performance power. We evaluated the ABM calibration by comparing model results from the “Business-as-usual” (BAU) scenario with historic data for key variables such as the number of active farms, agricultural area, or livestock numbers (Supplementary Figure SI7A and B), in line with the validation by results approach proposed by Troost and Berger (2015). To ensure transparency, we followed the ODD protocol (Grimm et al. 2006, 2010; Müller et al. 2013) to document model inputs and calibration.

## Results

### Extreme events

**Fig. 5:**
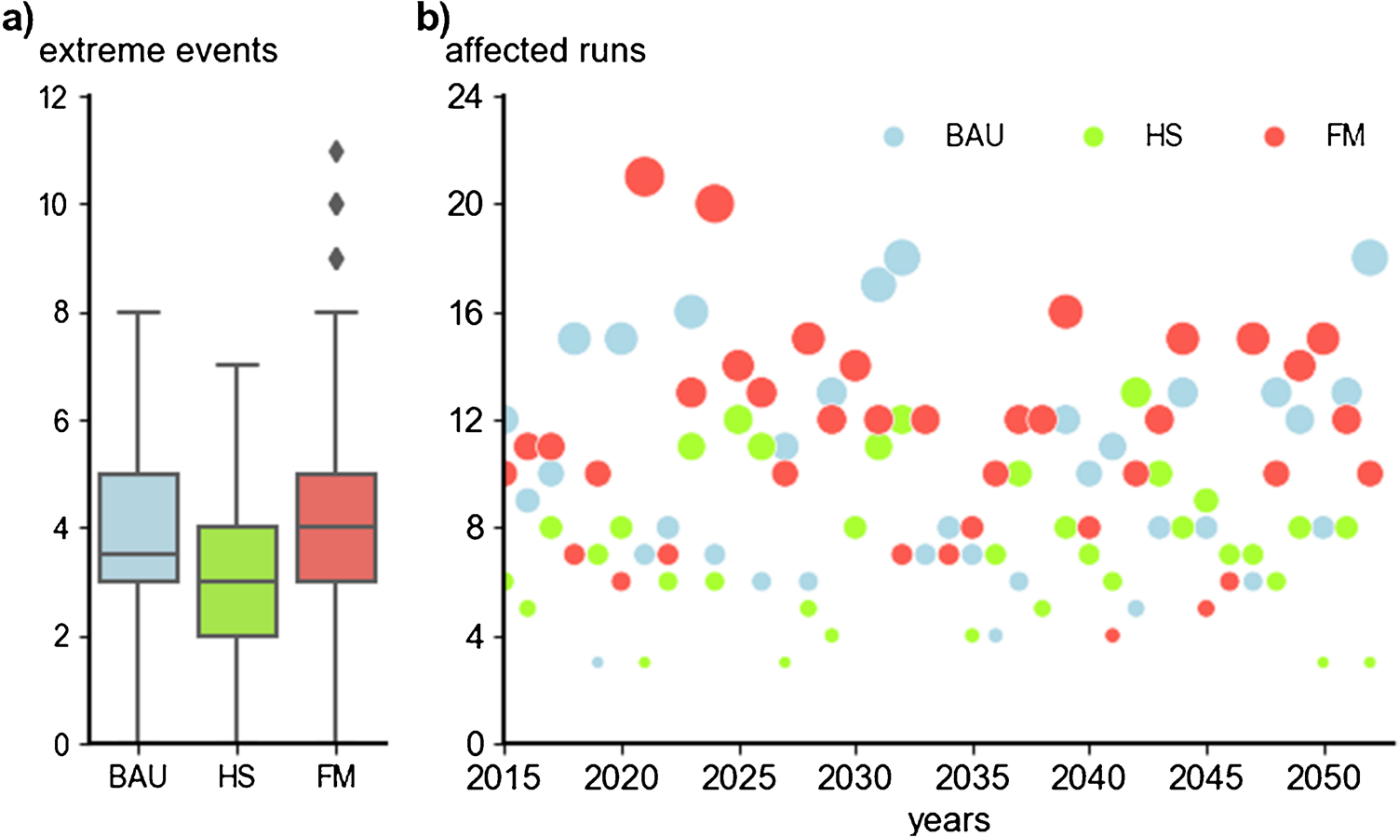
Comparison of Monte-Carlo (MC) results of extreme events. **a** Box-plots showing the distribution of extreme events with mean numbers of occurrence per run varying between 3 and 4. **b** Statistical overview of years affected by extreme events compared for all MC runs and scenarios; the size of the circles is used as indicator for the occurrence of extreme events (small=low, medium=intermediate, large=high). Scenarios assessed: business-as-usual (BAU), a sustainability-driven high subsidy (HS) and low-subsidy free market (FM) scenario.

The 300 simulation runs led to a total of 1,080 extreme events. The MC distribution of extreme events in Fig. 5a shows overlaps between scenarios. On average, each 37-year run involved 3.73 (BAU), 2.81 (HS), and 4.26 (FM) extreme events. In all scenarios, runs without extreme events occurred. This is contrasted by a maximum of 11 events in one run of the FM scenario (BAU, 8; HS, 7). The scenario comparison shows a broader variation with outliers (diamond shaped points) for the FM scenario. The years most affected by extreme events vary greatly among scenarios (Fig. 5b). While in the BAU scenario, the years 2032 and 2052 were most affected by extreme events in 18 runs; this number was lower for the HS, where the years 2025 and 2042 are hit in 12 and 13 runs. For the FM scenario, the extreme events occurred earlier, in 2021 and 2024, and in higher number with 21 and 20 runs. For each year and run, the model tracked the occurrence of extreme events and we reused these modeling outputs from the non-adaptive scenarios, as inputs for the adaptive scenarios. Having similar extreme events within (adaptive and non-adaptive) scenarios allowed us to study the effects of adaptation.

### Farms and areas

**Fig. 6:**
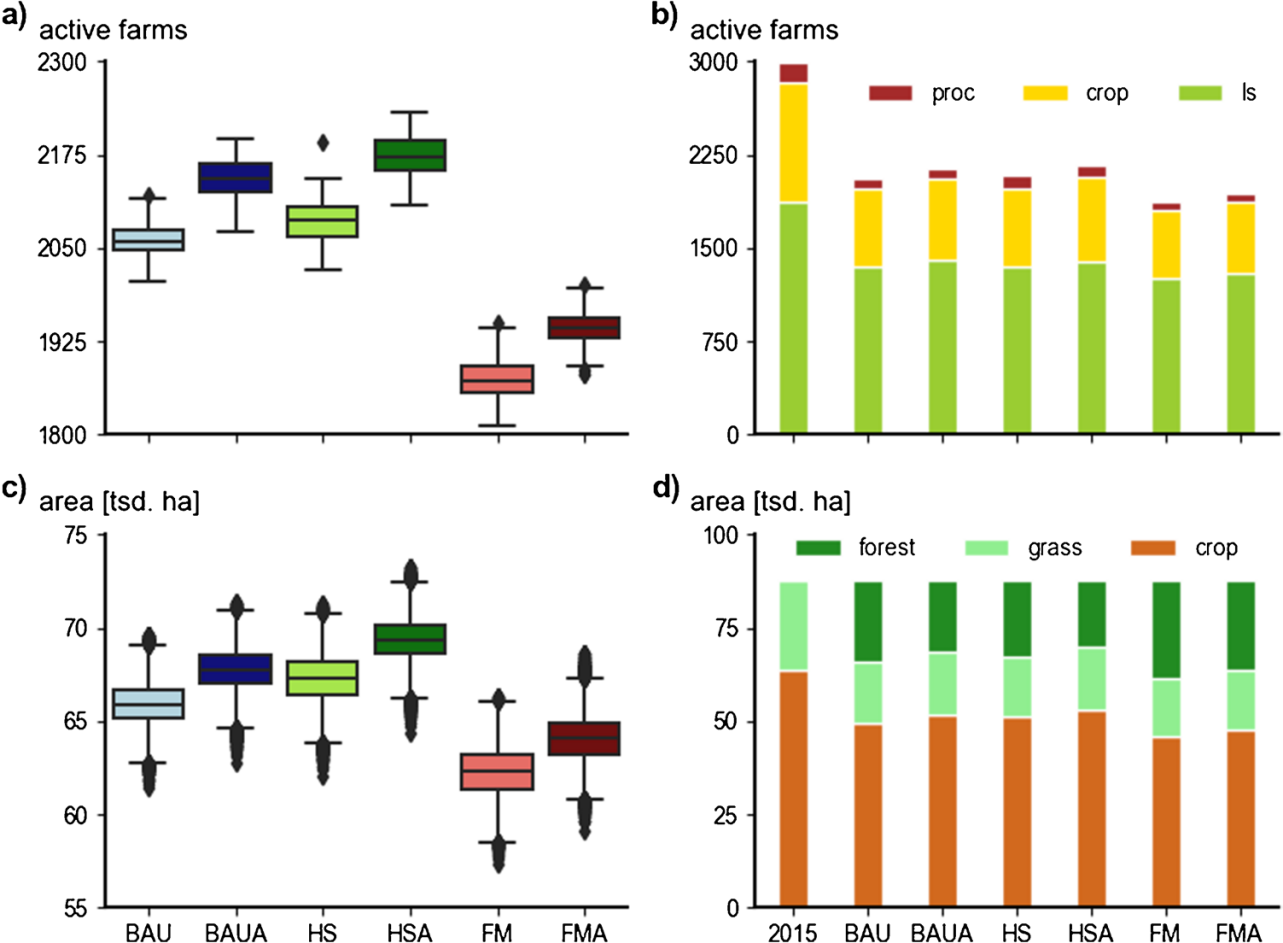
Comparison of the simulation results between scenario runs without adaptation (BAU, HS, FM) and adaptation scenarios (indicated by the suffix A in the scenario code: BAUA, HSA, and FMA). Distribution of the Monte Carlo (MC) results 2053 for (**a**) active farms and (**c**) agricultural area; comparison of initial 2015 numbers against 2053 means for (**b**) active farms split by farm type (proc=processing, crop=cash crop, ls= livestock) and (**d**) mean areas split by land-use type (forest, grass=grassland, crop=cropland). Scenarios assessed: Business-as-usual (BAU), a sustainability-driven high subsidy (HS), and low-subsidy free market (FM) scenario.

The mean across all runs and scenarios showed a decline from the initial number of 2990 active farms in 2015 to 2059 in 2053. In the BAU scenario, the number of active farms decreased by 31%, which was similar to the HS scenarios (2,085 farms, −30%). The FM scenario showed a stronger decline to (1873 farms, −37%) in 2053 (Fig. 6a). The results of the adaptive scenarios illustrated similar farm abandonment trends, but at lower rates, with 2141 (−28%) active farms in 2053 in BAUA, and comparable lower, respectively, higher numbers for HSA (2173; −27%) and FMA (1941; −35%). In bivariate comparison for 2053, the adaptive scenarios depicted significantly (*p*<0.01) higher values of about +4% in MC means of active farms. The split of mean active farms in 2053 by farm type (Fig. 6b) revealed higher scenario sensitivity for farm types, with numbers varying between −37% (HSA) and −61% (FM) for processing, −29% (HSA) and −39% (FM) for cash crop, and −25% (BAUA) and −33% (FM) livestock farms. The adaptation-related differences of mean active farms split by farm type varied from 3 (livestock farms) up to 6% (processing farms).

Corresponding to the decline of the numbers of farms, also a decline of agricultural area was detected with means ranging from 69,848 (HSA) to 61,568 ha (FM) in the year 2053 when compared to the initial 87,545 ha in 2015 (Fig. 6c). The mean decrease of agricultural areas and assumed subsequent forest transition of abandoned agricultural areas (Fig. 6d) was considerably lower in the BAU (−25%) and HS (−23%) scenarios than in the FM (−30%). Again, the adaptive scenarios depicted differences of about 4%, meaning fewer agricultural areas abandoned in 2053 and therefore also less forest transition. The decrease of cropland was smaller compared to grassland. Cropland declined by −17% (HSA) to −28% (FM), with higher cropland cultivation in the adaptive scenarios. Trends were similar in all scenarios, with stronger pronunciation for the FM scenarios. The scenario outcomes reflected the range of results for agricultural area, as the difference between highest (HSA) and lowest (FM) means of cultivated cropland in 2053 marks 11% of the total cropland area 2015. Grassland depicted less scenario variation with consistent decreases of about −32% to −35% until 2053, again with lower rates (∆3%) of abandonment in the adaptive scenarios (see Supplementary Table SI8). The highest area of grassland cultivated in 2053 was observed in the BAUA scenario. Compared to cropland, the range of results for grasslands was less diverse, with differences of about 6% of 2015 grassland area.

### Effects from adaptive management

**Fig. 7:**
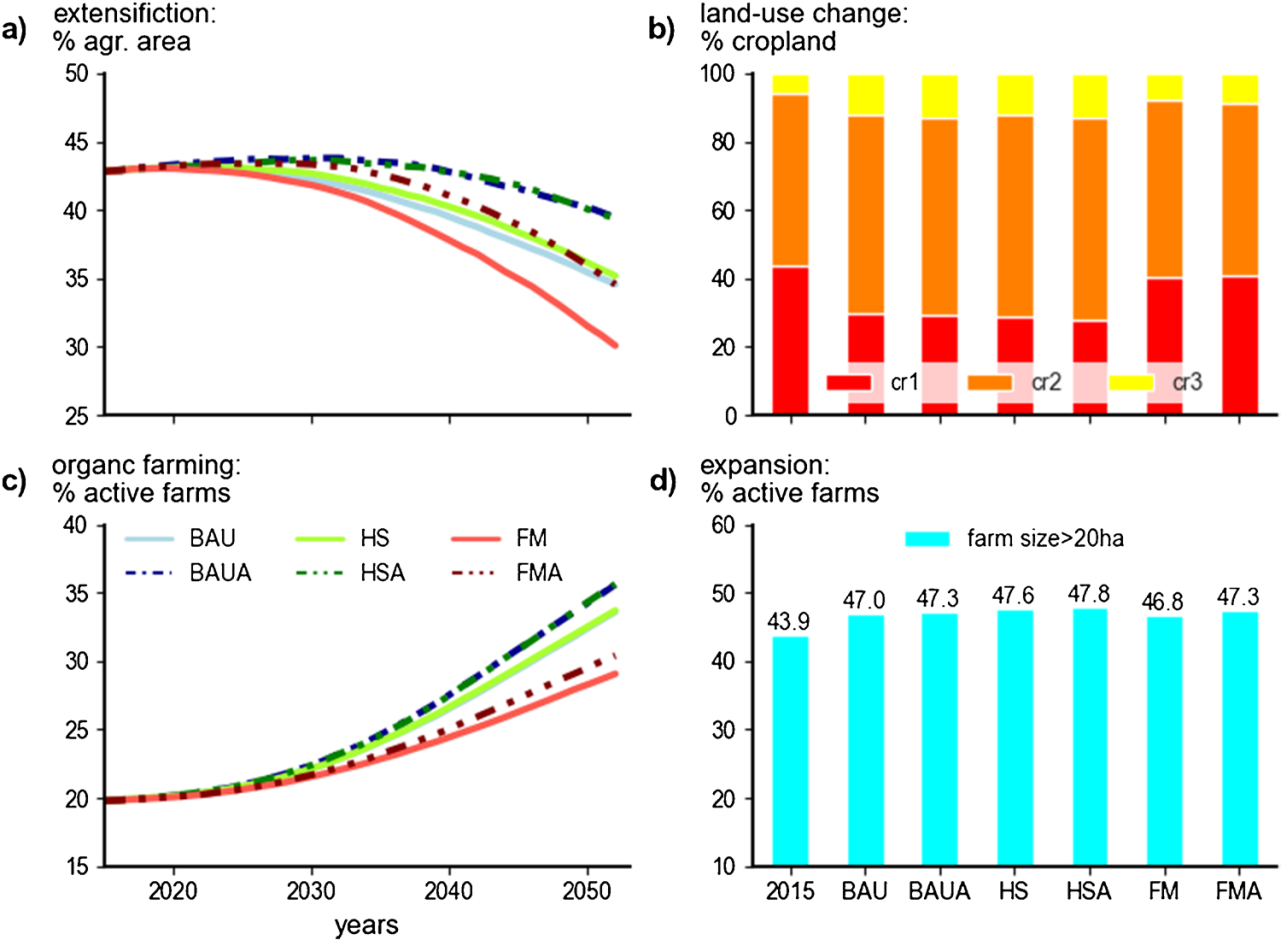
Cross comparison of four indicators related to the adaptation actions: (**a**) for extensification the mean development of shares of extensive (intensity level <3) areas; (**b**) for land-use change the mean cropland shares split by crop cycle in 2053; (**c**) for organic production the mean development of shares of organic farms; (**d**) for expansion the mean share of farms > 20ha in 2053 (with 20 ha being the Austrian average of agricultural area per farm in 2016). Scenarios assessed: business-as-usual (BAU), a sustainability-driven high subsidy (HS), and low-subsidy free market (FM) scenario; adaptation scenarios are indicated by the suffix A.

We used four specific indicators (Fig. 7a–d) to assess the impact of the adaptation measures *extensification*, *land-use change*, *organic farming*, and *expansion*. The development trajectories for the shares of extensive areas and the shares of organic farms in the adaptive scenarios highlight the influence of *extensification* and *organic farming* (Fig. 7a, c). Extensive areas ranged from 30% to 40% in 2053, with noticeably higher shares for all adaptive scenarios (Fig. 7a). The corresponding results of the FMA and BAU scenario underlined the effect of adaptation. Across all scenarios, increasing shares of organic farms until 2053 was a dominant future development trend (Fig. 7c). Compared to 2015, the proportion of organic farms nearly doubled for the BAU and HS scenarios until 2053, especially in the adaptation scenarios where this trend was more pronounced. Despite low, absolute numbers in the FM scenarios in 2053 (BAU, 545; BAUA, 590; see Supplementary Table SI8), nevertheless, the relative share of organic farms increased even in these scenarios.

In contrast, the comparison of the initial values of 2015 and with 2053 means for selected indicators showed marginal effects for the adaptation measures *land-use change* and *expansion* (Fig. 7b, d). The cropland shares among the crop cycles (cr1, cr2, cr3; see Supplementary Table SI1) revealed similar development for the BAU and HS scenarios with expanding shares for crop cycles 2 and 3, contrary to the FM scenarios with higher shares of cr1 in 2053 (Fig. 7b). The similarity between the adaptive and non-adaptive scenarios showed that the development of the crop cycles was primarily driven by the scenario conditions. These favored the cropping of soy (lead crop cr2) and rapeseed (lead crop cr3) in the BAU/HS scenarios and upholded the production of root crops (lead crop cr1) in the FM scenarios. The shares of farms with a farm size of more than 20ha remained merely stable among scenarios (Fig. 7d). While in 2015 about 44% of farms exceeded 20ha, this number increased slightly towards 46%–47% in 2053. Interestingly, the results depicted the highest farm sizes in the HS scenarios, compared to the lowest shares in the FM scenario, which indicated that the high subsidy volumes and prices allowed more farms to increase their farm size.

### Income and workload satisfaction

**Fig. 8:**
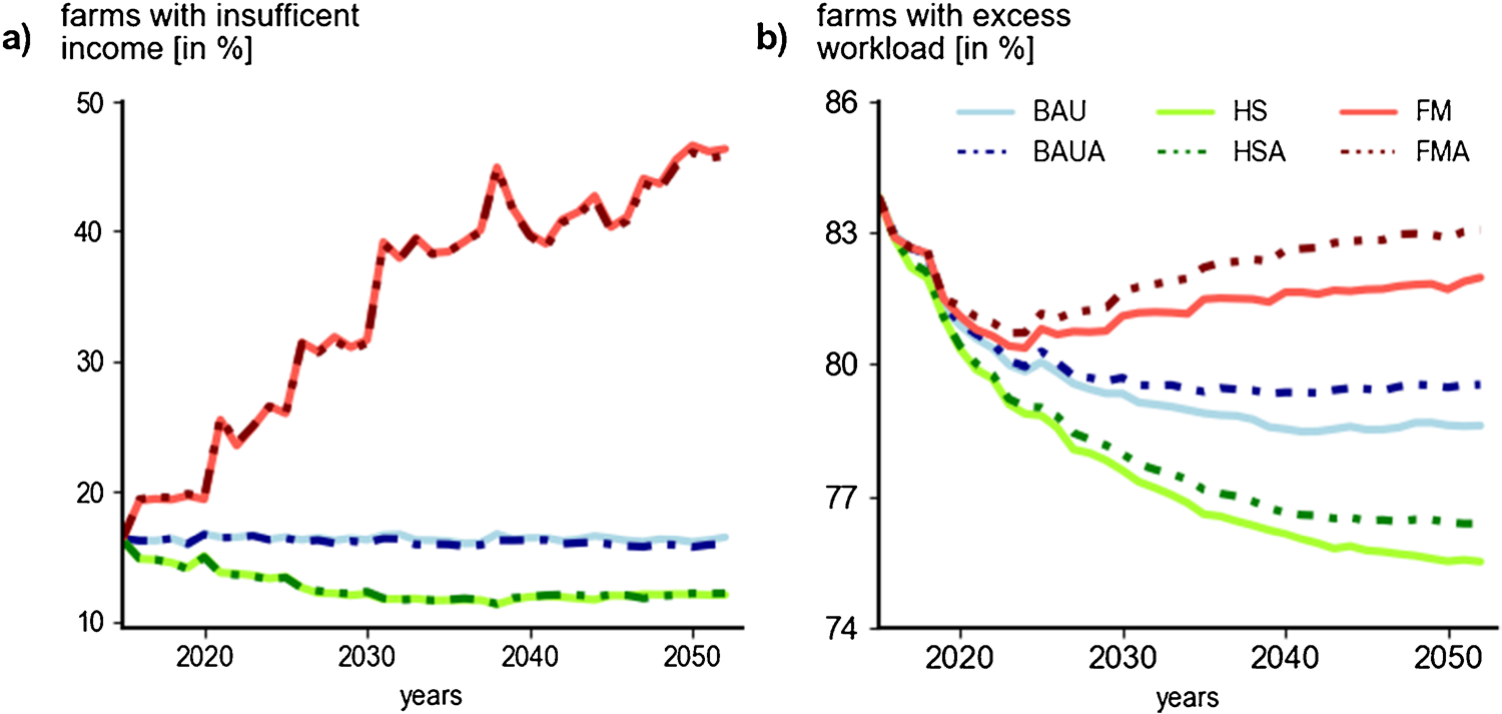
Development of shares of farms 2015–2053 with insufficient income (**a**), respectively, the share of farms excess workload (full-time workers (fte) working more than 1800 h/year) (**b**). Scenarios assessed: business-as-usual (BAU), a sustainability-driven high subsidy (HS) and low-subsidy free market (FM) scenario; adaptation scenarios are indicated by the suffix A.

The impact of learning on farmer satisfaction was represented by its impact on agricultural income and workload, with a positive effect for income (higher absolute numbers) and a negative effect for workload (Fig. 8a–b). We found that the scenario conditions were the decisive factor for the development of the mean share of farms with insufficient income (Fig. 8a). While in the BAU settings the rate of farms with insufficient income remained stable over time, the favorable conditions of the HS scenarios slightly decreased this rate. In contrast, in the FM scenarios, the mean rate of farms unsatisfied with income increased to nearly 50% by 2053.

The comparison of the development of the mean share of farms with excess workload showed similar scenario trends but consistently higher rates for the adaptive scenarios (Fig. 8b). Until about 2025 across scenarios, farms pursued a reduction of workload with decreasing shares of farms that work more than 1800 h/fte/year. From then onwards, the scenario conditions had a stronger effect, which was reflected in the continued declines under the favorable conditions in the HS scenarios, decelerated and stagnant development in the BAU scenarios, and a trend reversal with increasing numbers in the FM scenarios. In all adaptive scenarios, the trajectories depicted higher workloads per full-time equivalent for the period between 2025 and 2053; for the FM scenarios, this was already observed from 2020 onwards. In both graphs, the impact of extreme events was visible in the punctual spikes of the trajectories.

## Discussion

This paper investigates the impact of learning from extreme weather events on land-use decision-making and subsequently agricultural development. For this purpose, we integrated learning into the SECLAND ABM and simulated future trajectories for three scenarios for a rural region in eastern Austria. Despite the simulated, unilateral decline of active farms and agricultural area until 2053, the results showed that adaptation made a difference by buffering this evolution. However, its impact varied depending on the scenario conditions and increasing labor demand emerged as limiting factor for adaptation.

With the integration of learning in the SECLAND ABM, we build on the two essential factors of the decision-making process: the farming style symbolizing the intrinsic motivation of farmers, and well-being (a balance between income and workload). It is common in agent-based modeling to address risk-related adaptation of an agent through imitation or orientation within its social network (van Duinen et al. 2016; Amadou et al. 2018; Gawith et al. 2020; Zagaria et al. 2021; Marvuglia et al. 2022; Huber et al. 2022). However, this acknowledges only on the social aspect of learning, while the SECLAND ABM integrates learning as socio-ecological process that builds on the farming style and the occurrence of the extreme events. This model design brings two advantages. First, the decision to model varying degrees of inertia towards adaptation depending on the farming style integrates heterogeneity among the farmer population. Second, the increasing effect of extreme events on adaptation desire allows the consideration of gradual transition to adaptation but avoids conglomerated network effects.

While SECLANDs farm agents consider workload and income as decisive factors for their actions, farmers are commonly parameterized to seek profit maximization (Filatova et al. 2009; Schreinemachers and Berger 2011; Zimmermann et al. 2015; van Duinen et al. 2016; Amadou et al. 2018; Yang et al. 2020; Zagaria et al. 2021; Huber et al. 2022). Consequently, the consideration of non-financial aspects or limitations is underrepresented in the decision-making processes of farm agents. For a case study under climate change in New Zealand, Gawith et al. (2020) identified that socio-economic constraints on farmers’ decision-making resulted in considerably lower adaptation rates compared to pure profit maximization, and concluded that economic models underestimated the realistic cost of adaptation. Although adaptation had a positive effect on the number of active farms and agricultural area under cultivation in 2053, the SECLAND ABM depicted a realistic picture on (social and economic) costs and revealed that this did not translate to the well-being of farms. Income satisfaction was predominantly driven by socio-politic scenario conditions, and partially affected by the occurrence of extreme events but independent of adaptation. This confirms empirical analyses on Italian, Hungarian, Slovakian, and Swiss farmers (Severini et al. 2016; Brunner and Grêt-Regamey 2016; Bojnec and Fertő 2019), demonstrating that direct payments from the first pillar of the Common Agricultural Policy (CAP) increase and stabilize farm income by partially offsetting production and market volatilities (Lurette et al. 2020). Furthermore, our simulations highlighted workload as trade-off factor for the decision-making of farms. On the one hand, the already high initial burden presented a strong motive for farms to purse a reduction in their workload. Limitation in the production factors capital and labor represent important constrains for diversification of farms, especially concerning family farms and the seasonal peaks during harvest season (Bowman and Zilberman 2013). On the other hand, did adaptation result in a higher workload for farms, which is connected to the dominant adaptation measures organic farming and extensification, as organic and extensive farming (small-scale farming in general) are associated with higher labor demand then conventional or highly mechanized industrial farming (BAB 2021). This means that farms only managed to compensate environmental risks by increasing their individual workload.

### Adaptation strategies

We followed a participatory modeling approach that involved stakeholders into several phases of the project from data collection and scenario outline to the model definition (Hare 2011). On the one hand, participatory modeling can increase modeling performance and results (Gaube et al. 2009; Smetschka and Gaube 2020). On the other hand, it gives heterogenous stakeholders the opportunity to use the option space created by future scenarios as base for discussions and to compare their future management options (Carmona et al. 2013). Due to the COVID-19 pandemic, we had to adapt the planned stakeholder activities (adopt one online workshop followed by expert interviews instead of recurring in person meetings), which unfortunately had an impact on the involvement of local stakeholder. Nevertheless, we managed to capture essential topics in our exchanges with regional stakeholders. During the interviews, farmers mentioned price pressure, the need to expand production, and subsequently rising workload of farm workers as well as concerns about the negative impacts from recurring droughts on agricultural production in the study region. Furthermore, the discussion on future agricultural development during the stakeholder workshop revealed the desire for experiments and alternative support approaches as part of substantial reforms for the current agro-environmental framework to promote a fundamental transformation of agriculture.

We deducted four adaptation strategies *organic production*, *extensification*, *expansion*, and *land-use change* based on farm management strategies from the 20 interviews in the study region supplemented with additional 40 interviews with farmers in Austria and France. With the shift to organic and extensive production, farms can decrease the pressure on production quantity; additionally, higher prices can increase their gross margins (Resare Sahlin et al. 2022; Garmendia et al. 2022). As farms in this application of the SECLAND ABM only consider the agricultural income as satisfaction criteria, they cannot substitute farm income with off-farm work (Brunner and Grêt-Regamey 2016). Instead, they can decrease their external input costs (e.g., fertilizer, machinery) through an extensification strategy. Additionally, through organic production, extensification, and by expansion of their farm size, farms can increase the inflow of subsidies from both pillar 1 (direct payments) and pillar 2 (rural and structural development) of the CAP framework. *Land-use change* gives farms a chance to change their agricultural products, and switch between crops and crops cycles, to adapt to a changing climatic and economic conditions (Wheeler et al. 2013; Marvuglia et al. 2022). We did not consider the strategic reduction of farm area (Wheeler et al. 2013). The exchange with local stakeholders revealed early on that irrigation systems are not a viable solution for the region mainly for economic (small-scale agricultural structure with scattered fields) and legal reasons (conflicts with the water regulation framework, already existing water scarcity). As a result, this study offered an alternative approach towards climate change adaptation, compared to the generally in literature proposed introduction of irrigation systems (Wheeler et al. 2013; van Duinen et al. 2016; Amadou et al. 2018; Yang et al. 2020; Zagaria et al. 2021).

The simulation results showed varying degrees of effects from the adaptation measures. Future cropping patterns depicted a shift from sugar beet towards rapeseed and soy cultivation, mainly influenced by scenario settings. In Europe, rapeseed is an essential crop for the production of biodiesel (van Duinen et al. 2016), which is as substitute for fossil fuels, especially important in sustainability scenarios. Independent of the scenario, we observed an increase of soy cultivation in 2053. The production of domestic, high-quality GMO-free soy has become a lucrative market for European farmers, which competes with the cultivation of established crops in terms of cropland demand. Especially for organic production, soy is attractive due to its ability to fix atmospheric nitrogen in organic crop rotations; moreover, it generates good income due to its high price (Fogelberg and Recknagel 2017).

Despite a trend towards farm enlargement within EU countries (Jurkėnaitė and Baležentis 2020), the simulations suggested only a marginal increase for the share of farms exceeding the 2016 Austrian average farm size of 20 ha (Grüner Bericht 2021). However, there are other factors to consider for farms in terms of productivity and profitability than just possible economies of scale from a larger farm size. An empirical analysis from Australia depicts that, especially for smaller farms, access to advanced production technology plays a decisive role in increasing farm productivity (Sheng et al. 2015). For European countries, the farming type also plays a decisive role, as diverse and mixed farms oppose farm size growth, while specialized crop and livestock farms showed the highest growth rates in the last decade (Jurkėnaitė and Baležentis 2020). On the other hand, the consideration of workload threshold in the decision-making is a limiting factor in the expansion of farm size. The analysis of EU Farm Accountancy Data Network (FADN) highlights the ability to rely on family labor capacity as key determinant of farm profitability for small and medium farms (Kryszak et al. 2021).

The results revealed the shift to organic production and decrease of production intensity as most decisive actions for land-use and farm management. The dominant trend of increasing organic farms, which was even more pronounced in the adaptive versions, aligned well with historic developments. Since Austria’s accession into the EU in 1995, the share of organic farms has been rising strongly. Austria reached the EU goal of 25% of organic agricultural areas in 2030, already in 2020 with a share of 26.4%, placing it among the EU countries with the highest shares of organic farming (Kummer et al. 2021). Additionally, several studies identified organic farmers as young, educated, better informed, and less risk averse then conventional famers (Koesling et al. 2004; Flaten et al. 2005; Tzouramani et al. 2014; Schattman et al. 2016; Läpple et al. 2016; Mitter et al. 2019), meaning that they have higher sensibility for climate change and are more willing to adapt their production; therefore, ultimately more of them will exist in 2053.

The comparison of the results for agricultural area showed that losses of extensive land occur in all scenarios, although the extent of the decline differed between the scenarios. We observed varying land sharing and land sparing strategies (Grass et al. 2021), as the reduction of subsidies in the FM scenarios reduced total farmed area, but the remainder was cultivated at higher intensity. By contrast, higher subsidies in the HS scenarios kept more agricultural area under cultivation, but this land was farmed with lower intensity. Irrespective of scenario narrative, adaptation led to higher shares of extensive areas and generally more agricultural area in 2053. This has two implications for the sustainability of farm management. First, the reduction of external inputs such as fertilizers and pesticides serves as a risk management strategy (Ahsan and Roth 2010; Tzouramani et al. 2014; Schattman et al. 2016) that reduces a farmers’ exposure to fluctuating production costs (Serra and Duncan 2016; Bojnec and Fertő 2019). Second, organic and extensive farming reduces these input costs but increases the revenues (prices, subsidies) simultaneously. In addition, organic farmers consider environmentally friendly agricultural practices to be effective measures for climate change adaptation (Schattman et al. 2016; Mitter et al. 2019), which was confirmed by regional farmers during our interviews (Fessler 2021; Petz 2021).

### From ex-post to ex-ante adaptation

Our results projected the decline of about one-third of the initially active farms accompanied by the loss of one-quarter of the initial agricultural area up until the year 2053. Although a decline in agricultural production seems inevitable, the scenario comparison showed that the setting of the external framework conditions can slow down or accelerate this trend. Independent of the scenario conditions, our results highlighted the positive effect of adaptation on the number of active farms as well as agricultural area. This corresponds with findings of Zagaria et al. (2021) and van Duinen et al. (2016), who based the adaptation mechanism on farmers’ perception of drought risk for adaptation, for case studies in Italy and the Netherlands. Whether adaptation measures incorporate the transition of an agricultural production system or not, modeling frameworks that build learning and adaptation on climate change-related extreme events, first need a negative impact to create an ex-post response that changes farmers’ decision-making. The earlier an extreme event occurs the sooner it triggers adaptive decisions of farmers, and the longer it influences their decision-making. On the other hand, every extreme event has a negative impact on farm income and farmers’ satisfaction, as farmers have to keep investing in damage control limiting their options. We deliberately decided to apply slight variations in the stochastic extreme event functions to capture these offsetting effects, which can be seen in the compilation of the scenario results. In the HS scenario, there was less incentive for adaptation due to the low frequency of extreme events. In the FM scenario, the high rate and severity of extreme events in combination with declining subsidies decreased the efficiency of adaptation actions. The apparent difference between the results with and without adaptation in the BAU scenarios highlighted efficient adaptation based on the interplay between subsidy volume and extreme event frequency. Therefore, if adaptation is implemented as reaction to a negative event, any circumstances that lower farmers’ distress (e.g., compensation payments, “mild” climate change) also reduce their perceived risk and consequently their desire for adaptation. In addition, there is the temporal aspect of adaptation, which have a greater effect at lower costs, the earlier they are implemented (Brunner and Grêt-Regamey 2016).

For a shift towards ex-ante adaptation, the negative stimulus must be substituted by an alternative driver. One way to overcome this dilemma provides the focus on positive incentives as motivator for the implementation of adaptation measures ex-ante. Such a change in perspective would dissolve the constraint of climate-related risk perception and broaden the group of potentially adopting farmers. The scenario comparison provides valuable implications for socio-political measures that could guide such as result-based payments approach. The success and efficiency of the CAP measures and its reforms have been broadly discussed and especially criticized for the shortcoming in support for environment- and climate-friendly practices, despite its major financial volume (Serra and Duncan 2016; Pe’er et al. 2019, 2020; Scown et al. 2020). How effectiveness of the CAP can be increased in a target-oriented manner has yet to be defined (Scown et al. 2020). To add to this discussion, we investigate the potential impact of socio-political measures on adaptation decision-making and agricultural development in the “high subsidy” and the “free market” scenarios. The comparison of our results indicates that financial incentives alone are insufficient: despite the significant increase in subsidies in the HS scenarios, the superior results from the adaptive BAU scenario depict comparable outcomes in active farms and agricultural area and illustrate the potential positive effect of adaptation on agro-structural development.

On the other hand, synergies to existing farm management practices play a role in the adoption of agro-environmental and climate measures of farmers (Fanchone et al. 2022). The findings of this study are in line with the EU farm-to-fork strategy that builds on organic and low-input farming with the aim to strengthen regional production and consumption (Kummer et al. 2021). Our analysis also highlights workload as limiting factor for the decision-making of farms as their already high workload increased further with successful adaptation. This crystallizes workload reduction as an essential factor to incentivize management shifts. Socio-political measures that provide temporal and seasonal farm labor support have the potential to increase the sustainability of farms, not only farm profitability but also the social well-being of farm households.

For this first application of learning in the SECLAND ABM, we concentrated solely on the integration of learning and on aggregated dynamics on study region level. The analysis of spatial heterogeneity as driver for adaptation (Delay et al. 2015) was beyond the scope of this paper, as we focused on the comparison of results with and without adaptation. Nevertheless, we plan to address the implications of learning in a heterogenous study region in future research.

## Conclusion

European farmers are embedded in complex socio-ecological systems. Their decision-making is influenced not only by restrictions set by farm production capacity, but they also have to comply with the guidelines lined out by the national and international subsidy schemes. Additionally, in the upcoming decades, farmers will face the challenge to adapt to changing climatic conditions that have long-term effects as well as abrupt extreme events that affect agricultural production.

For this study, we integrated learning through climate change adaptation into the SECLAND ABM. In a novel approach, we linked the effects of extreme events (environmental) and value-based characteristics (social and economic) on farmers’ decision-making to integrate learning as socio-ecological process into the model. This allowed us to use the particular advantage of agent-based modeling and simulate the effects of learning on socio-economic farm structure development as well as its spatial impacts on land-use dynamics across diverse future scenarios.

From the scenario analysis, we conclude that adaptation has a positive impact on both the number of active farms and the agricultural area in 2053. However, successful adaptation does not necessarily mean a shift to an irrigation system, which is an infeasible solution in small-scaled, or grassland-dominated farming systems. We identified organic and extensive farming as successful and well-established adaptation strategies to climate change. Our results also depicted that adaptation was accompanied by an additional increase of the already high workload for farmers. Furthermore, the comparison of the results against the socio-politic framework conditions revealed the limited scope of financial incentives.

We identified the need for agricultural subsidy schemes that increase the scope of action for farms, by supporting them in workload reduction in the long run. To increase the resilience of farms and achieve successful long-term shifts towards sustainable development paths in agriculture, individual and societal efforts must complement each other. Future directives of the CAP should therefore focus not only on economic and ecological but equally on social objectives in order to create sustainable framework conditions for European farmers.

## Supplementary Information

Below is the link to the electronic supplementary material.

Supplementary file1 (DOCX 194 KB)

Supplementary file2 (XLSX 21 KB)

## Data Availability

The agent-based module (ABM) is written in Netlogo 6.0.2. The open-source software is accessible on https://ccl.northwestern.edu/netlogo/index.shtml. The model code is not publicly available but a thoroughly description can be found in the supplementary material. A request for use for the Austrian IACS data can be made with the ministry for agriculture, regions, and tourism via https://dafne.at/. The socio-economic data are publicly available from Statistik Austria https://www.statistik.at/ and Bundesanstalt für Agrarwirtschaft und Bergbauernfragen BAB - Deckungsbeiträge und Kalkulationsdaten (agrarforschung.at).
