# Supplementary material for: Effects of extreme events on land-use-related decisions of farmers in Eastern Austria: the role of learning
Source: Agron Sustain Dev. 2023 May 12;43(3):39. doi: 10.1007/s13593-023-00890-z (PMC10176289; doi:10.1007/s13593-023-00890-z)
Supplement: Supplementary file 1 — Supplementary file1 (DOCX 194 KB) [file 13593_2023_890_MOESM1_ESM.docx]

**Effects of extreme events on land-use related decisions of farmers in Eastern Austria: the role of learning**

*Claudine Egger^1*^, Andreas Mayer^1^, Bastian Bertsch-Hörmann^1^, Christoph Plutzar^2^, Stefan Schindler^2,3^, Peter Tramberend^2^, Helmut Haberl^1^ and Veronika Gaube^1^*

*^1^ Institute of Social Ecology, Department of Economics and Social Sciences, University of Natural Resources and Life Sciences, Vienna, Schottenfeldgasse 29, Vienna 1070, Austria*

*^2^ Environment Agency Austria, Spittelauer Lände 5, Vienna 1090, Austria*

*^3^ Community Ecology and Conservation, Faculty of Environmental Sciences, Community Ecology and Conservation research group, Kamýcká 129, CZ-165 00 Prague 6, Czech Republic.*

*Corresponding author: [claudine.egger@boku.ac.at](mailto:claudine.egger@boku.ac.at)

**Keywords**: agent-based modelling; learning; scenario analysis; adaptation; Austria; agriculture

**Methods**

**Table SI1:** Overview land-use classes and characteristics

| **LU class** | **crop type** | **slope** | **crop cycle** | **intensity** | **yields** |
| --- | --- | --- | --- | --- | --- |
| **Cropland** | corn | unique slope class | cr1: corn, cereals, root crops, cereals, (field forage*) cr2: corn, cereals, soy, cereals, (field forage*) cr3: corn, cereals, rape, (field forage*) *org crop cycles end with field forage | IL 0: area without cultivation (rent market)  IL 1-5: differing work requirement and yields/ gross margins depending on the farm IL and production system (conv/org) | IL 1: 0.9* IL2 IL 2: 0.9* IL3 IL 3: AWI calculator conventional/organic (for Lower Austria)  IL 4: 1.1* IL3 conv/ 1.05*IL3 org IL 5: 1.1* IL4 conv/ 1.05*IL4 org |
|  | cereals |  | cr1, cr2, cr3 |  |  |
|  | field forage  (broad bean) |  | cr1, cr2, cr3 org |  |  |
|  | soybean |  | cr2 |  |  |
|  | winter oilseed rape |  | cr3 |  |  |
|  | root crops |  | cr1 |  |  |
|  | fallow |  | no rotation |  | no yields |
| **Grassland** | grassland meadow | s1: <25° s2: 25°-35°  s3: > 35° | no rotation |  | IL 1: 1 cut/ 0.75 conv/ 0.5 org (lu/ha) IL 2: 2 cuts/ 1.25 conv/ 1 org (lu/ha) IL 3: 3 cuts/ 1.5 conv/ 1.25 org (lu/ha) IL 4: 4 cuts/ 2.0 conv/ 1.5 org (lu/ha) IL 5: 5 cuts/ 2.5 conv/2 org (lu/ha) see Table SI5 for slope class differentiation |
|  | grassland pasture |  | no rotation |  |  |
|  | mountain pastures | unique slope class | no rotation | IL 0: rent market IL 1 -5: floored at IL 1 | IL 1: 1 cut/ 0.75 lu conv/ 0.5 lu org |
| **Forest** | deciduous | no slope class | no rotation | no intensity | no yields |
|  | coniferous |  |  |  |  |
| **Abbreviations:**  org = organic production  conv = conventional production  cuts = number of cuts on meadows  lu = livestock units  lu/ha = number of livestock units per ha (pasture)  IL = intensity level | | | | | |

**Table SI2:** ODD protocol of the SECLAND ABM

*Due to the size of the table, we attached it in a separate excel file (see ODD_Table.xslx).*

**Input data**

**Table SI3**: Area input data

| **Variable** | **Definition** | **Source** |
| --- | --- | --- |
| Primary key | unique patch identifier linking to IACS parcels | Generated |
| Area size | Patch area [ha] | IACS GIS |
| Forest | Defines natural succession deciduous and coniferous forest | GIS generated |
| Patch class | Area classifier that distinguishes cropland, grassland, fallow and forest areas | generated |
| Crop culture | Model specific crop cultures build from patch class, intensity, and slope | Assigned |
| Cropland suitability | Assigns the suitability of transforming grassland patches into cropland | GIS generated |
| Slope class | Slope classes that divide grassland patches into three different groups based on its the slope | GIS generated |
| Maximum area | Binary variable that marks the largest field of a farm, which serves as farm location in the ABM | Assigned |
| Farm type | Assigned farming type: cropland, processing, and livestock farms | IACS, generated |
| Farm unit | Unique farm identifier | IACS GIS |
| Farming intensity | 5 intensity levels:  1: cropland: avg. organic yields  grassland: 1 cut (meadow), 1 lu (pasture)  2: cropland: avg. FADN yields -10%  grassland: 2 cuts (meadow), 1,25 lu (pasture)  3: cropland: avg. FADN yields  grassland: 3 cuts (meadow), 1,5 lu (pasture)  4: cropland: avg. FADN yields +10%  grassland 4 cuts (meadow), 1,75 lu (pasture)  5: cropland: highest yields FADN farms  grassland: 5 cuts(meadow), 2 lu (pasture) | Based on 2014 yields from FADN farms (“Buchführungs-betriebe”),  IACS-subsidies guidelines,  Water Framework Directive BGBl. Nr. 215/1959, 1959) |
| Rent regions | Divides study area into different rent regions for patch renting | GIS generated |

**Table SI4:** Farm input data

| **Variable** | **Definition** | **Source** |
| --- | --- | --- |
| Farming style | Reflects farmers belief system: yield optimizer, traditionalist, support optimizer, innovative, idealist | Randomly assigned, based on Schmitzberger et al. (2005) |
| Age of farm manager | Age of the farm manager | Assigned based on statistical data for Upper Austria West/ Lower Austria and Burgenland East |
| Family workers | Initial number of farm workers that is calculated on initial agricultural labor demand multiplied by random value [0.9, 1.1] | Calculated |
| Maximum working hours | Total farm workers * work endowment (1800) [hrs] | Calculated |
| Decision counter | Counter that counts the years until the next possible action (3 years) | Initially: randomly assigned number between 1 and 3 |
| Activity status | Binary variable to mark active farms (1) from inactive farms (0) | Assigned |
| Farm type | Cash crop (without livestock), processing (pig fattening/suckler production) and (cattle) livestock farms (milk/meat) | Assigned |
| Farm unit | Unique farm identifier | IACS analysis |
| Farming intensity | Intensity level 0: rent market (patches)  Intensity levels 1-5 for farms and farmed (patches) | Assigned based on IACS analysis |
| Rent regions | Divides study area into different rent regions for patch renting | GIS defined for IACS map |

**Table SI5:** External input data

| **Variable** | **Definition** | **Source** |
| --- | --- | --- |
| Agricultural labor demand | Working hours needed for a specific production system, crop culture and farming intensity | <https://idb.awi.bmlfuw.gv.at/> |
| Gross margins | Gross margins calculated specifically for production system, intensity and crop culture for cropland, respectively livestock density (lu/ha) for grasslands | <https://idb.awi.bmlfuw.gv.at/>, book keeping farms |
| Yields | Cropland study site West:  IL 1: 0.9* IL2  IL 2: region specific from book keeping farms  IL 3: AWI calculator conv/org (for Upper Austria)  IL 4: region specific from book keeping farms but higher than IL3  IL 5: 1,1*IL4 conv/ 1,05*IL4 org  Cropland study site East:  IL 1: 0.9* IL2  IL 2: 0.9* IL3  IL 3: AWI calculator conv/org (for Lower Austria)  IL 4: 1.1* IL3 conv/ 1.05*IL3 org  IL 5: 1.1* IL4 conv/ 1.05*IL4 org  Grasslands West/East (*slope class1, **s2, ***s3):  IL 1 conv: 1 cut/ 0,75*,0,64**,0,55***lu/ha  IL 2 conv: 2 cuts/ 1,25*,1,14**,1,05***lu/ha  IL 3 conv: 3 cuts/ 1,5*,1,41**,1,33***lu/ha  IL 4 conv: 4 cuts/ 2*,1,86**,1,75***lu/ha  IL 5 conv: 5 cuts/ 2,5*,2,37**,2,07***lu/ha  IL 1 org: 1 cut/ 0,5*,0,41**,0,35*** lu/ha  IL 2 org: 2 cuts / 1*,0,89**,0,82*** lu/ha  IL 3 org: 3 cuts/ 1,25*,1,15**,1,09*** lu/ha  IL 4 org: 4 cuts/ 1,5*,1,37**,1,29*** lu/ha  IL 5 org: 5 cuts/2*,1,8**,1,77*** lu/ha | <https://idb.awi.bmlfuw.gv.at/>, book keeping farms |
| Subsidies | Subsidies computed for crop culture and farming intensity | Calculated based on the analysis of reported subsidy values for both study regions in IACS data |
| Wage for non-agricultural work | Hourly wage of non-agricultural income | Based on data from Statistik Austria for Lower Austria and Burgenland |
| Existential minimum income | Threshold for farmer’s satisfaction with income | Based on reported data in  „Grüner Bericht Niederösterreich 2017“ |
| Maximum working hours | Threshold for farmer’s satisfaction with workload | defined |
| Agricultural wage | Wage for external agricultural laborers | Machine cooperation |
| Initial farm workers | Initial number of farm workers per farm | Calculated based on IACS data |

**Table SI6**: Overview stakeholder process

| Approach | Topics | Details | Participants | Date |
| --- | --- | --- | --- | --- |
| Semi-structured interviews | Influence of intrinsic and extrinsic factors (framework conditions) on land-use decision making | High workload of family farm workers Role of subsidies Climate change/Extreme events (less spring rain, increasing heat waves and droughts, alien (plant) species, erosion) Irrigation systems: water scarcity concern, economic viability, scattered fields, new/deeper wells as alternative | 20 local farmers  (9 cattle livestock, 11 cash crop farmer) | Summer 2020 |
| Stakeholder workshop | Past, current and future agricultural trends in the study region | Past/current trends: Dependance on subsidy schemes to uphold small-scale agriculture, expansion (of farmland/livestock numbers), specialization and intensification Climate change: drought significant issue, erosion protection, dry soil, water storage in soil, drought resistant plants  Irrigation systems: in conflict with water regulations, water scarcity and small-scale agriculture  Regional economic development  Future trends: digitalization, social aspects become more essential, new crops, irrigation extremely costly Transformation: substantial reform of framework conditions, need for experiments and alternative subsidy approaches | 10 participants, i.e.,  farmers, local agricultural chambers, regional decision-maker, NGO’s, researchers | July 2020 |
| Expert interviews | In-depth discussion of the topics raised during the stakeholder workshop | Deepening and follow-up discussion of the discussion/topics/trends from the stakeholder workshop,  Scenario outlines: climate/yield/subsidy/price/regional changes | 2 regional agricultural experts | November 2020 |
| Stakeholder workshop | Presentation and discussion of results | Presentation and discussion of the results for land-use and ecosystem service indicators | 12 participants, i.e. regional decision-makers, local agricultural chambers, researchers | March 2023 |
| Final symposium | Final project presentation | Synthesis of the results for land-use and ecosystem service indicators | 7 (registered) participants, i.e. researchers, agricultural chambers, NGO, public administration | April 2023 |

**Model evaluation:**

**
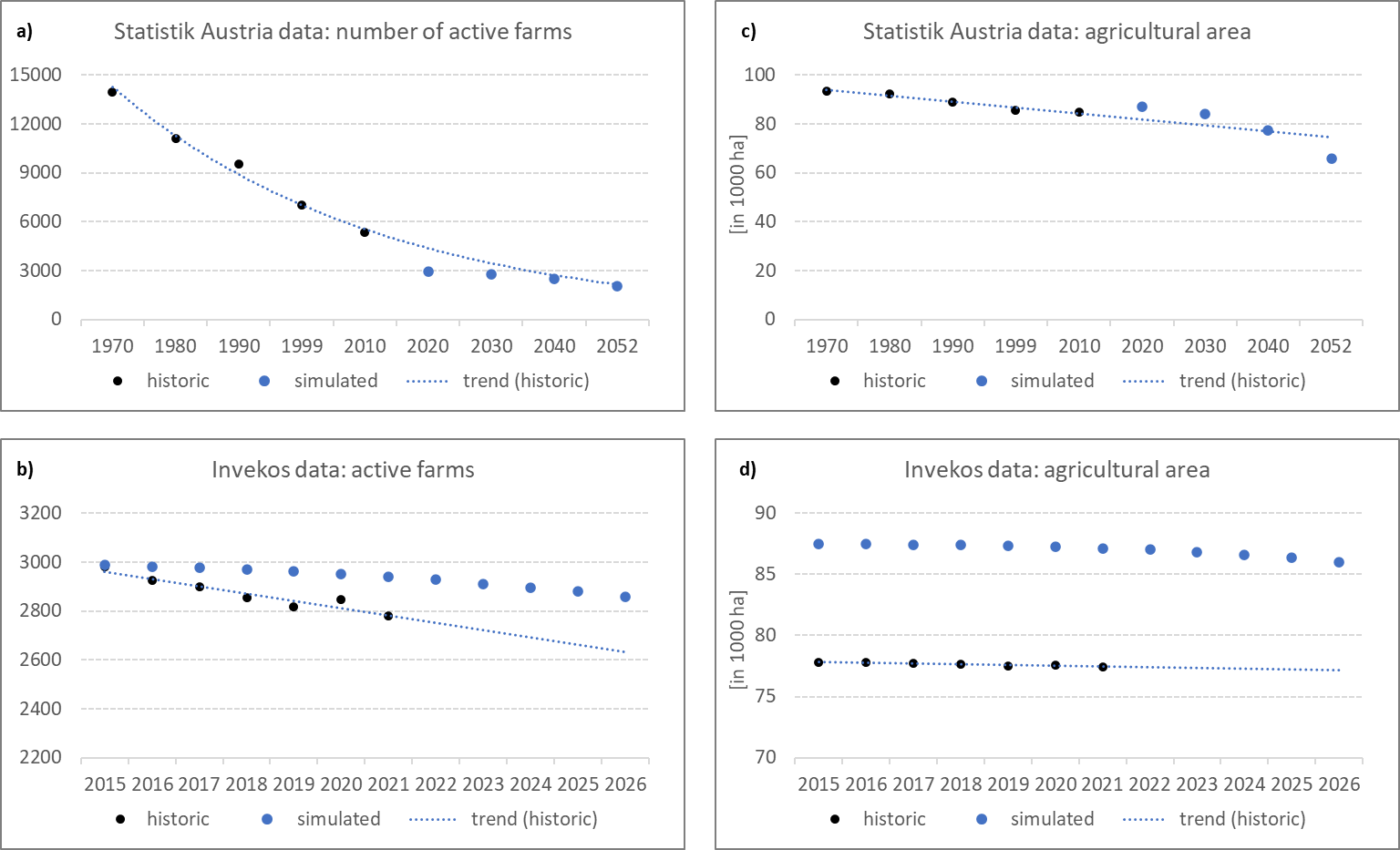
**

**Figure SI7A:** Comparison of historic and simulated data for active farms (a and c) and agricultural area (c and d): As the data sets were not directly comparable (e.g., the Invekos data are subsets of the data reported by Statistik Austria; differences due to system boundaries), we used historic data from Statistik Austria (a and b) and Invekos (b and d) to validate the modelling results.

**
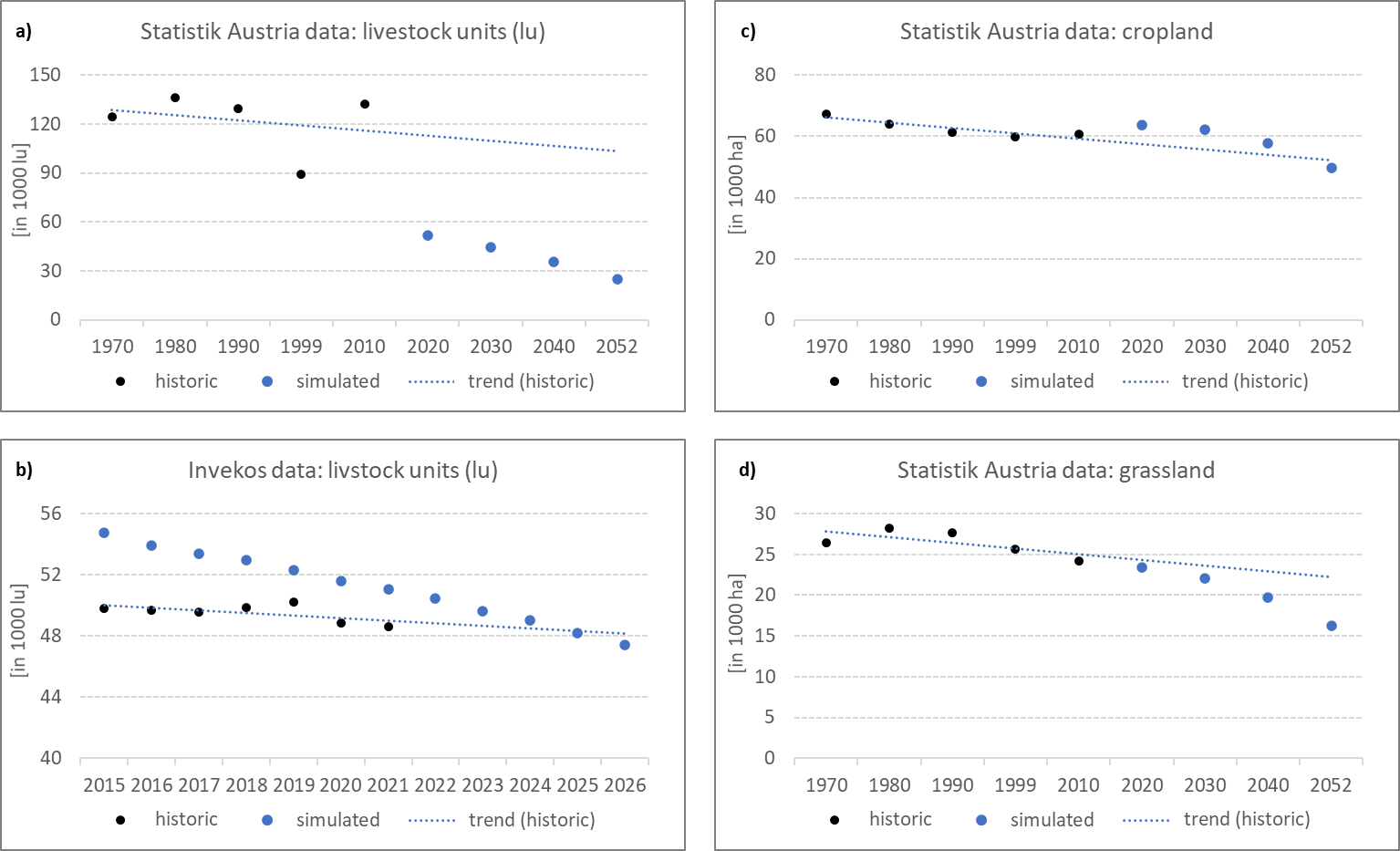
**

**Figure SI7B:** Comparison of historic and simulated data for cropland, grassland and livestock units: As the data sets were not directly comparable (e.g., the Invekos data are subsets of the data reported by Statistik Austria; differences due to system boundaries); we used historic data from Statistik Austria (a) and Invekos (b) to validate the modelling results for livestock units (lu); in addition we compared simulated grassland (c) and cropland (d) data against historic data from Statistik Austria.

**Results**

**Table SI8:** Results overview: comparison between 2015 and 2053, scenarios and model versions

|  | | **2015** | **2053** | | | | | | | | |
| --- | --- | --- | --- | --- | --- | --- | --- | --- | --- | --- | --- |
|  |  |  | **BAU** | **BAUA** | *% Δ adapt. BAU* | **HS** | **HSA** | *% Δ adapt. HS* | **FM** | **FMA** | *% Δ adapt. FM* |
|  |  |  | **mean** | **mean** |  | **mean** | **mean** |  | **mean** | **mean** |  |
| **number of active farms** | **total** | 2,990 | 2,059 | 2,141 | 4% | 2,085 | 2,173 | 4% | 1,873 | 1,941 | 4% |
|  | *% diff. 2015* | | -31% | -28% |  | -30% | -27% |  | -37% | -35% |  |
|  | **cash crop** | 954 | 626 | 659 | 5% | 638 | 675 | 6% | 556 | 580 | 4% |
|  | *% diff. 2015* | | -34% | -31% |  | -33% | -29% |  | -42% | -39% |  |
|  | **processing** | 162 | 75 | 77 | 3% | 98 | 102 | 4% | 63 | 67 | 6% |
|  | *% diff. 2015* | | -54% | -52% |  | -40% | -37% |  | -61% | -59% |  |
|  | **livestock** | 1,874 | 1,357 | 1,404 | 3% | 1,348 | 1,395 | 3% | 1,253 | 1,293 | 3% |
|  | *% diff. 2015* | | -28% | -25% |  | -28% | -26% |  | -33% | -31% |  |
|  | **organic farms** | 591 | 692 | 762 | 10% | 703 | 774 | 10% | 545 | 590 | 8% |
|  | *% diff. 2015* | | 17% | 29% |  | 19% | 31% |  | -8% | 0% |  |
| **area in ha** | **agricultural** | 87,545 | 65,893 | 68,382 | 4% | 67,068 | 69,848 | 4% | 61,568 | 63,919 | 4% |
|  | *% diff. 2015* | | -25% | -22% |  | -23% | -20% |  | -30% | -27% |  |
|  | **crops** | 63,847 | 49,690 | 51,525 | 4% | 51,040 | 53,135 | 4% | 46,053 | 47,811 | 4% |
|  | *% diff. 2015* | | -22% | -19% |  | -20% | -17% |  | -28% | -25% |  |
|  | **grassland** | 23,699 | 16,202 | 16,855 | 4% | 16,026 | 16,712 | 4% | 15,513 | 16,106 | 4% |
|  | *% diff. 2015* | | -32% | -29% |  | -32% | -29% |  | -35% | -32% |  |
|  | **forest** |  | 21,653 | 19,164 | -11% | 20,478 | 17,698 | -14% | 25,978 | 23,627 | -9% |
